# Discriminatory performance of mid-upper arm circumference for identifying thin and severely thin adolescents: a secondary data analysis using Comprehensive National Nutrition Survey

**DOI:** 10.3126/nje.v11i2.33926

**Published:** 2021-06-30

**Authors:** Nitika

**Affiliations:** 1 Independent Public Health Consultant, India

**Keywords:** Anthropometry, Body mass index, ROC curve, Malnutrition, Nutritional status

## Abstract

**Background:**

Timely identification of adolescents with undernutrition is of utmost importance, and recently, mid-upper circumference (MUAC) had been considered as an alternative to body mass index (BMI) and BMI for age z-score (BAZ) for its screening. However, little is known about the MUAC cut-offs, specific to age and sex. The study was planned to assess the discriminatory performance of MUAC in identifying thin and severely thin adolescents and estimating age specific MUAC cut-offs, separately for males and females, against BAZ as the gold standard.

**Methods:**

The Comprehensive National Nutrition Survey (CNNS), India data was used for this analysis. The Receiver Operating Characteristic curve (ROC), area under curve (AUC), and Youden Index were used to estimate MUAC cut-off values for thin (BAZ < -2) and severely thin (BAZ < -3) adolescents. The current analysis was done on 31471 adolescents.

**Results:**

The MUAC cut-offs for identifying thin adolescents were: for 10-14 years – 19.2/19.4 cm, for 15-19 years – 22.9/21.7 cm for males and females respectively; and for severe thinness were: for 10-14 years – 18.4/18.3 cm, for 15-19 years – 21.9/20.2 cm for males/females. For thinness, the cut-off varied between 17.4-24.5 cm (for 10-19 years) among males, and for females, it varied between 17.5-20.9 cm (for 10-19 years). For severe thinness, MUAC cut-off ranged between 16.4-23.7 cm (for 10-19 years) among males, and for females, between 17.3-20.7 cm (for 10-19 years).

**Conclusion:**

MUAC, a easy to use measure demonstrated an equivalent diagnostic performance for the identification of thinness and severe thinness against BAZ. Thus, age- and sex-specific cut-offs could be considered for screening thin and severely thin adolescents.

## Introduction

Adequate nutritional status during adolescence is of utmost priority [1]. In 2007, WHO classified thinness as BMI for age z-score (BAZ) < -2, and severe thinness as BAZ < -3 [2]. Globally, the prevalence of thinness was 8.4 % among females and 12.4 % among males in 2016 [3]. In India, the prevalence of thinness was quite high, 22.7 % among females, and 30.7 % among males in 2016 [3]. Undernutrition during adolescence adversely affects school performance, work productivity, timing of puberty, and catch-up growth [4-6]. Thus, timely identification of adolescents with undernutrition is of utmost importance.

BMI and BAZ, commonly used measures to identify undernutrition, are quite challenging in resource limited settings [3]. BMI assessment requires accurate measurement of height and weight with the help of calibrated instruments, and high chances of error creeping-in if the instruments are not calibrated properly or not kept on properly levelled surface. Further, health care staffs must compute BMI using weight and height followed by its z-scores using growth chart. Given this, use of a more practical, handy, and easy to use measure could be considered for the assessment of undernutrition in resource limited settings.

Mid upper arm circumference (MUAC) has the potential for assessing undernutrition [7]. Currently, global standards are available for assessment of severe acute malnutrition (SAM) in under-5 age group [8]. A study by Sethi et al from eastern part of India, had proposed the age-specific cut-offs for screening thin and severely thin females in 10-19-year age group [9]. Another study from Ethiopia had proposed cut-offs for 15-19-year age group for screening thinness [10]. However, there is limited data pertaining to the age-specific cut-offs separately for males and females using a nationally representative sample.

With this background, the study was planned to assess the diagnostic performance of MUAC in identifying thin and severely thin adolescents and estimating the age-specific and age-group specific (early adolescence: 10-14 years, and late adolescence: 15-19 years) cut-off values for MUAC among adolescents, separately for males and females compared against BAZ as the gold standard.

## Methodology

### Study design and participants

Comprehensive national nutrition survey (CNNS) a collaborative effort of UNICEF and Population Council, under the leadership of the Ministry of Health and Family Welfare, Government of India. was conducted during 2016-18. Rural and urban sampling units were selected using a multi-stage, stratified, probability proportional to size sampling approach. Then, from rural and urban sampling units, only the households with children or adolescents were selected. From each household, one child/adolescent was selected.

### Data Collection

Computer Assisted Personal Interviewing (CAPI) method using mini laptop and data collection forms for anthropometric measures were used for data collection. The pretested structured questionnaire translated into 20 state-specific languages was used to collect demographic information. The complete details of the CNNS survey has been published elsewhere [11]. CNNS unit level data has been used for the analysis.

Trained anthropometrist working in team (of two) recorded anthropometric measurements in the household of each participant. The standardization exercises were conducted prior to beginning of data collection. All anthropometric equipments were calibrated daily.

MUAC was measured using standard fiberglass tapes on the right arm after identifying the midpoint of the upper arm and applying standard pressure while measuring the circumference. Standing height was measured using three-piece wooden height boards with legs extended and head set in the Frankfurt position. Body weight was assessed using digital SECA weighing scale in light clothes. During data collection, height and MUAC were assessed twice, and mean was used further, and weight was measured once. MUAC and height were measured to the nearest 0.1 cm, and weight to the nearest 0.1 kg. Weight and mean height measures were used to compute the BMI, using the formula: weight (kg)/height (m)2 and z-scores were calculated using the WHO International growth reference data [2]. BMI z-score less than -2 SD, and less than -3 SD was considered the criteria for thin and severely thin adolescents, respectively.

Technical error of measurement (TEM), for assessing intra- and inter-observer error, was computed for height and MUAC, and published elsewhere [11].

### Inclusion criteria

Adolescents having information for MUAC, BMI, and BAZ were included for the analysis.

### Exclusion criteria

**Figure 1.**
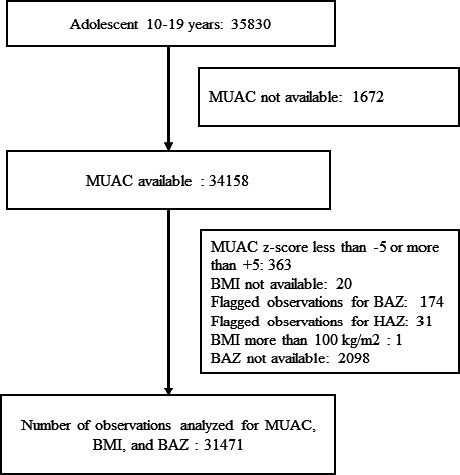
Number of adolescents included in analysis and reasons for exclusion

Afterwards, observations with flagged z-scores (for BAZ or HAZ) were excluded. (Figure 1)

### Outcome Variable

Mid upper arm circumference measured for the adolescents.

### Explanatory Variable

Age, gender, BMI and BAZ assessments done for the adolescents.

### Ethical clearance

The CNNS proposal was approved by Postgraduate Institute for Medical Education and Research, Chandigarh, India, and Population Council, New York Ethics Committee. De-identified version of dataset has been used for the current analysis, and hence, ethical approval was not required.

### Data management and statistical analysis

The analysis was done using Stata 15.0 (StataCorp, College Station, TX, USA) and statistical software R version 3.3.3 (The R Foundation for Statistical Computing, Vienna, Austria). Descriptive statistics, mean and standard deviation (SD) for quantitative variables, were estimated for summarizing participants characteristics. For estimating the prevalence of thin and severely thin adolescents, weighted analysis using national weights was done. Pearson correlation coefficient, a measure of strength of linear relationship, was estimated for MUAC and BAZ. Receiver operating characteristic (ROC) curve analysis was done to assess the discriminatory performance and determining the cut-off of MUAC for correctly identifying thin and severely thin adolescents. Thinness and severe thinness defined based on BMI-z score was used as the reference or gold-standard, against which performance of MUAC to assess the body thinness has been evaluated.

Area under ROC (AUC), was calculated for MUAC for each combination of young/late adolescent and separately for males and females. An AUC of 1 reflects a diagnostic test with perfect ability to differentiate between two categories, however difficult to achieve in practical scenarios. More the AUC value near to 1, more is the curve closer to the left upper corner, and more is the discriminatory ability. Further, a test with AUC value more than 0.8 is considered as good [12].

Additionally, to determine the MUAC cut-off point for correctly differentiating thin/normal weight adolescents, Youden Index, J (sensitivity + specificity – 1), was calculated, using “cutpt” command in Stata [13, 14]. J equals 1 for a perfect diagnostic test and 0 for a poor diagnostic test. Gender, age specific, and age-group specific (young adolescence: 10-14 years and older adolescence: 15-19 years) MUAC cut-offs were estimated for identifying thin and severely thin adolescents.

## Results

The current analysis was done using information on 31471 (15313 females, and 16158 males) adolescents, and reason for exclusion is detailed in Figure 1. The mean age was 14.0 years (SD 2.6). The mean (SD) for age, BMI, BAZ, height, MUAC, and weight is provided in Table 1. Among adolescents, the prevalence (weighted) of thinness and severe thinness was 23.9 % (95% CI: 23.5, 24.5 %) and 6.4 % (95% CI: 6.1, 6.7 %) respectively.

**Figure 2.**
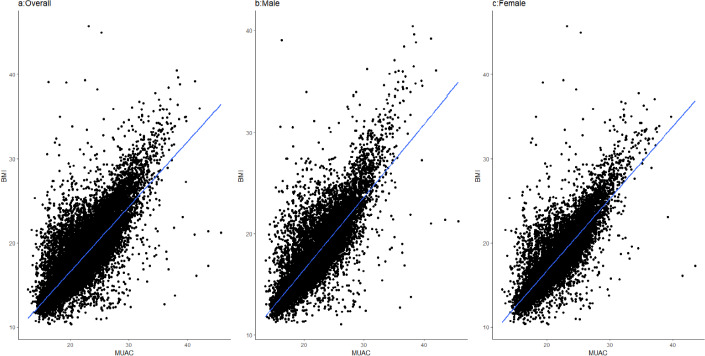
Scatter plot showing the correlation between body mass index (BMI) and mid-upper arm circumference (MUAC) a) Overall; b) Male: c) Female

There was significant correlation (p value <0.001) between measurements of MUAC v/s BMI (0.81) and MUAC v/s BAZ (0.63) (Figure 2).

**Figure 3.**
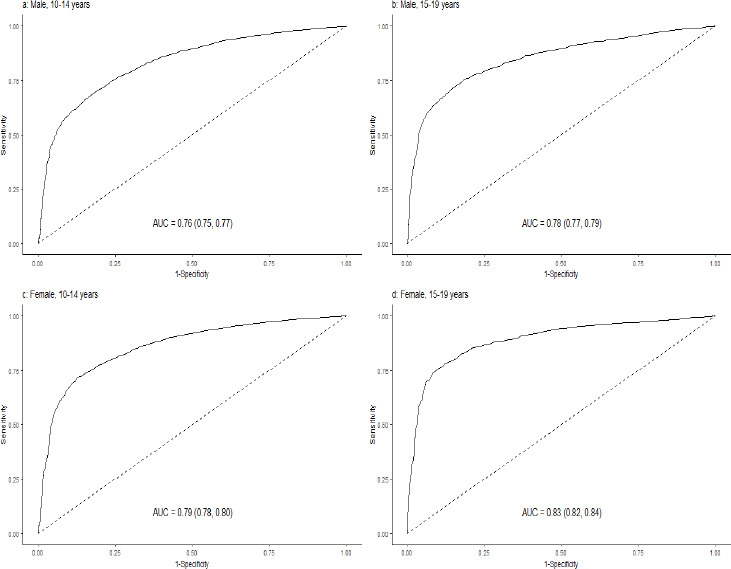
ROC depicting MUAC performance for identifying thinness among a) 10-14 years male; b) 15-19 years males; c) 10-14 years female; and d) 15-19 years female

**Figure 4.**
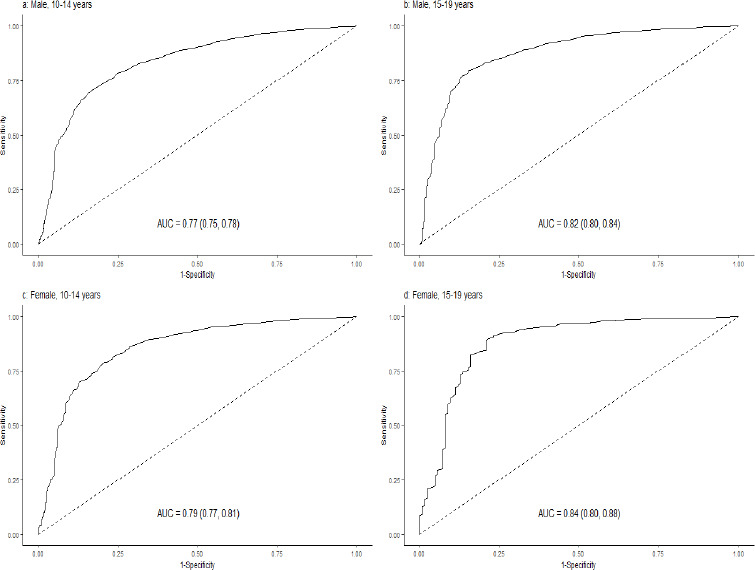
ROC curve depicting MUAC performance for identifying severe thinness among a) 10-14 years male; b) 15-19 years males; c) 10-14 years female; and d) 15-19 years female

Figure 3 depicts the ROC and AUC for MUAC to correctly identify thinness among males and females during early and late adolescence against the gold standard/reference based on BAZ. The AUC ranged between 0.76-0.83. Similarly, figure 4 depicts for severe thinness, with AUC ranging between 0.77-0.84.

Figure 4 - ROC curve depicts MUAC performance for identifying severe thinness among a) 10-14 years male; b) 15-19 years males; c) 10-14 years female; and d) 15-19 years female.

### Age specific MUAC cut-offs

Overall, for 10-19 years, based on Youden index, MUAC cut-off for thinness was 21.9 cm (males) and 20.4 cm (females), respectively. For males, the cut-off for thinness was 19.2 cm (10-14 years) and 22.9 cm (15-19 years). For females, the MUAC cut-off for thinness was 19.4 cm (10-14 years) and 21.7 cm (15-19 years). Table 2 summarizes the age specific MUAC cut-offs for identifying thin adolescents along with the diagnostic accuracy measures. For males, the cut-off varied between 17.4-24.5 cm (for 10-19 years) among males, and for females, it varied between 17.5-20.9 cm (for 10-19 years). The sensitivity and specificity ranged between 68.9-91.3 %.

Overall, for 10-19 years, the MUAC cut-off for severe thinness was 20.5 cm (males) and 19,4 cm (females). For males, cut-off for severe thinness was 18.4 cm (10-14 years) and 21.9 cm (15-19 years). For females, the MUAC cut-off for severe thinness was 18.3 cm (10-14 years) and 20.2 cm (15-19 years). Table 3 summarizes the age specific MUAC cut-offs for identifying severely thin adolescents along with the diagnostic accuracy measures. between 16.4-23.7 cm (for 10-19 years) among males, and for females, it ranged between 17.3-20.7 cm (for 10-19 years). The sensitivity and specificity ranged between 73.1-100 %.

## Discussion

The study has important findings regarding age- and sex-specific MUAC cut-offs for the identification of thin and severely thin adolescents (defined based on BAZ). The MUAC correlated well with BAZ, thus possibility of its applicability for screening of thin/severely thin adolescents. Furthermore, MUAC had good AUC values for age-specific cut-offs, suggesting the equivalent diagnostic performance in detecting thin and severely thin adolescents, as compared to BAZ.

### Correlation coefficient

The study found a significant correlation coefficient of 0.69-0.70 between MUAC and BAZ, and this is in coherence with the previous study findings [7, 9, 15-18].

### Area under curve

In the present study, AUC ranged between 0.77-0.93, signifying good-excellent diagnostic accuracy of MUAC cut-offs against BAZ defined thinness/severe thinness. Similarly, a study from India reported AUC value ranging between 0.82-0.97 for age specific MUAC cut-offs in identifying thin and severely thin female adolescents [9]. Another study from Ethiopia conducted among 15-19 years adolescents reported an AUC of 0.91 for correct identification of thinness [10]. These findings point towards considering MUAC as an alternative to BAZ in identification of thin/severely thin adolescents in resource-constrained settings.

### Age-specific and sex-specific MUAC cut-offs

The age-group MUAC cut-off to identify thinness were: for 10-14 years – 19.2/19.4 cm, for 15-19 years – 22.9/21.7 cm, for males and females respectively. Study by Sethi et al proposed the cut-off of 19.4 cm (10-14 years) and 21.6 cm (15-19 years) for females, like the present study [9]. In a study by Sisay et al, the proposed cut-offs for 15-19 years adolescents were 23.2 (males) and 22.6 cm (females), slightly different from the present study, might be due to the racial differences in body built, thus, pointing towards having country-specific cut-offs for adolescents [10,19].

Further, the proportion of adolescents correctly classified using MUAC cut-offs were in general more for late adolescents compared to early adolescents, possibly due to less variation in cut-offs for late adolescents as the growth is stable, similar to the findings from a previous study [9].

The age-specific cut-offs and age-group specific cut-off (specifically for early and late adolescents) have been estimated in the present study. However, there is considerable differences in these two, thus age-specific cut-offs should be preferred over age-group specific cut-off. For example, for thinness, the cut-off for young adolescent (10-14 years) females was 19.4 cm and for a 10-year-old female, it was 17.5 cm. Thus, using a cut-off of 19.4 cm for a 10-year-old female would classify a normal weight adolescent as thin. However, in certain situation where age is not known, age-group specific (10-14 years or 15-19 years) MUAC cut-offs could be considered.

Additionally, in field settings taking into consideration the feasibility of measurement of MUAC, it will facilitate early detection of thinness/severe thinness, followed by timely corrective measures. The adolescents having the MUAC below the cut-off would need further interventions, however, it is outside the scope of current manuscript.

MUAC assessment is undoubtedly, a relatively simple and easy measure requiring less time, in contrast to BMI assessment requiring stadiometer and weighing scale. Although, for the correct assessment of MUAC devoid of any measurement error, will require health staffs training for ensuring that it is done accurately and measuring tape is held with proper tension, not too loose nor too tight. Since health staffs generally assess MUAC among under-fives, it should not be a problem among adolescents. The colour coded tapes are used for under-fives, similarly colour coded tapes could be considered for adolescents to rapidly screen undernutrition among adolescents.

From policy perspective, and further integration in adolescent health programs, MUAC could be a potential option for correct identification of thinness and severe thinness. MUAC is a relatively inexpensive, as it only requires a measuring tape, and can be easily used in field settings, and in schools. Further, the results can be easily understood by caregivers and adolescents.

## Strength of the study

Firstly, the MUAC cut-off estimates are based on sample which is representative of entire nation. Secondly, MUAC and BMI were assessed using standardized field staffs and equipment’s, with strict quality control and monitoring in the CNNS. Third and most importantly, the age-specific, and age-group specific MUAC cut-offs have been proposed, separately for males and females for both thinness and severe thinness. This holds importance as the body composition of adolescents change with age, moreover, there was weak correlation between MUAC and age.

## Limitation of the study

The MUAC and BMI/BAZ were not assessed against a third indicator, this should not be a major limitation as BMI and BAZ are commonly used measure to detect undernutrition.

## Conclusion

MUAC, a easy to use measure demonstrated an equivalent diagnostic performance for the identification of thinness and severe thinness against BAZ. Thus, age specific MUAC cut-offs, separately for males and females could be used to screen thin and severely thin adolescents in India and similar settings. Further, effective integration of MUAC for screening in adolescent health program will need some modifications and careful planning, options like availability of colour coded tapes or MUAC charts could be considered.

### Future scope of the study

The incorporation of MUAC cut-offs in national health programmes will facilitate early detection of undernutrition among adolescents. However, it will require operational research regarding the best method for incorporating MUAC cut-offs in adolescent health programmes. Moreover, at global level it will help in initiating a discussion around having a simpler measure for screening thinness among adolescents.

### What is already known on this topic

Age-specific MUAC cut-offs for females to screen thin and severely thin 10-19 years adolescents, based on sample from two states in India.

### What this adds

This study provides a complete picture of age specific MUAC cut-offs for screening thin and severely thin adolescents using a nationally representative sample for both males and females.

## Figures and Tables

**Table 1.** Anthropometrics of adolescents included in the analysis (n=31471)

	Male (n = 16158) Mean ± SD	Female (n = 15313) Mean ± SD	Total (n = 31471) Mean ± SD
**Age, y**	13.9 ± 2.6	14.0 ± 2.6	14.0 ± 2.6
**Height, cm**	152.8 ± 13.9	147.9 ± 9.6	154.4 ± 12.3
**Weight, kg**	42.6 ± 12.9	40.4 ± 10.3	41.5 ± 11.8
**BMI, kg/m**^2^	17.8 ±3.3	18.3 ± 3.4	18.0 ± 3.4
**BAZ**	-0.9 ±1.4	-0.7 ± 1.2	-0.8 ± 1.3
**MUAC, cm**	21.9 ±3.7	21.8 ± 3.3	21.9 ± 3.5

**Table 2.** ROC curve analysis of MUAC cut-off values among adolescent for thinness

Age, y	Area under curve (95% CI^a^)	Cut-off value (cm)	Youden Index, J	Sensitivity (%)	Specificity (%)	PPV (%)	NPV (%)	Correctly classified (%)
**Males**								
**10**	0.81 (0.79, 0.83)	17.4	0.62	84.9	76.3	50.7	94.6	78.2
**11**	0.82 (0.79, 0.84)	18.1	0.64	85.7	78.3	55.4	94.6	80.1
**12**	0.81 (0.79, 0.83)	18.6	0.62	81.4	79.8	58.7	92.4	80.2
**13**	0.83 (0.81, 0.85)	19.8	0.66	89.6	76.2	55.0	95.7	79.5
**14**	0.83 (0.81, 0.85)	20.9	0.66	90.3	75.2	50.4	96.5	78.5
**15**	0.81 (0.79, 0.83)	21.9	0.63	88.7	73.9	48.6	95.9	77.1
**16**	0.82 (0.79, 0.84)	22.9	0.64	91.3	72.3	47.0	96.9	76.3
**17**	0.81 (0.79, 0.83)	23.6	0.63	90.9	70.7	43.8	96.9	74.7
**18**	0.82 (0.79, 0.84)	23.8	0.63	88.0	75.1	46.2	96.3	77.6
**19**	0.81 (0.75, 0.87)	24.5	0.61	89.5	71.8	40.5	96.9	74.9
**10-14**	0.76 (0.75, 0.77)	19.2	0.51	82.2	68.9	45.2	92.6	72.1
**15-19**	0.78 (0.77, 0.79)	22.9	0.57	81.9	75.1	46.0	94.1	76.5
**Females**								
**10**	0.79 (0.77, 0.82)	17.5	0.59	80.5	78.0	50.1	93.6	78.6
**11**	0.81 (0.79, 0.84)	18.2	0.64	82.9	79.9	53.3	94.4	80.6
**12**	0.83 (0.81, 086)	19.1	0.67	86.5	80.6	50.0	96.4	81.7
**13**	0.85 (0.82, 0.87)	19.4	0.70	82.5	87.3	55.4	96.3	86.5
**14**	0.84 (0.81, 0.87)	20.2	0.69	82.2	85.9	47.2	96.9	85.4
**15**	0.81 (0.79, 0.84)	20.8	0.64	79.4	83.6	40.3	96.7	83.1
**16**	0.84 (0.81, 0.87)	21.3	0.69	82.7	85.4	40.5	97.6	85.1
**17**	0.84 (0.81, 0.86)	21.9	0.68	90.9	76.9	33.5	98.5	78.5
**18**	0.85 (0.82, 0.87)	21.9	0.69	88.4	80.9	34.3	98.4	81.7
**19**	0.87 (0.80, 0.94)	20.9	0.75	83.3	91.3	61.0	97.1	90.2
**10-14**	0.79 (0.78, 0.80)	19.4	0.59	87.7	70.9	40.1	96.3	73.9
**15-19**	0.83 (0.82, 0.84)	21.7	0.66	87.9	77.8	33.5	98.1	78.9

^a^ CI = confidence interval

**Table 3.** ROC curve analysis of MUAC cut-off values among adolescent for severe thinness

Age, y	Area under curve (95% CI^a^)	Cut-off value (cm)	Youden Index, J	Sensitivity (%)	Specificity (%)	PPV (%)	NPV (%)	Correctly classified (%)
**Males**								
**10**	0.81 (0.76, 0.85)	16.4	0.61	75.7	85.4	23.8	98.3	84.9
**11**	0.81 (0.78, 0.84)	17.6	0.62	85.5	76.7	24.8	98.3	77.4
**12**	0.83 (0.80, 0.86)	18.1	0.66	87.6	78.1	27.8	98.5	78.9
**13**	0.85 (0.82, 0.88)	18.8	0.70	89.1	80.6	27.5	98.9	81.3
**14**	0.83 (0.79, 0.86)	19.9	0.67	86.5	78.9	24.3	98.7	79.5
**15**	0.82 (0.79, 0.86)	20.1	0.65	76.1	88.8	34.8	97.9	87.9
**16**	0.85 (0.81, 0.89)	20.9	0.70	82.9	87.3	27.3	98.9	87.0
**17**	0.86 (0.82, 0.90)	22.2	0.74	89.7	83.2	17.3	99.5	83.4
**18**	0.84 (0.79, 0.89)	22.6	0.69	83.8	84.3	17.7	99.2	84.3
**19**	0.77 (0.58, 0.97)	23.7	0.74	80.0	74.8	7.0	99.4	74.9
**10-14**	0.77 (0.75, 0.78)	18.4	0.54	82.1	71.6	19.0	98.0	72.4
**15-19**	0.82 (0.80, 0.84)	21.9	0.64	84.4	79.8	18.3	99.0	80.0
**Females**								
**10**	0.77 (0.73, 0.82)	17.3	0.55	82.2	73.1	17.0	98.4	73.6
**11**	0.81 (0.77, 0.85)	17.2	0.64	76.1	86.7	29.2	98.1	85.9
**12**	0.84 (0.79, 0.89)	17.9	0.69	79.8	88.8	26.2	98.9	88.4
**13**	0.85 (0.81, 0.89)	18.9	0.71	84.6	85.6	21.9	99.1	85.5
**14**	0.86 (0.82, 0.91)	19.6	0.73	87.0	85.6	17.0	99.5	85.6
**15**	0.85 (0.79, 0.91)	19.8	0.70	81.4	88.9	14.9	99.5	88.8
**16**	0.82 (0.75, 0.89)	20.2	0.66	73.2	90.4	15.6	99.3	90.0
**17**	0.91 (0.83, 0.98)	20.0	0.82	88.9	93.1	13.2	99.9	93.0
**18**	0.84 (0.74, 0.93)	20.7	0.67	78.9	88.1	7.7	99.7	88.0
**19**	0.93 (0.90, 0.95)	20.7	0.85	1.00	85.2	11.4	100.0	85.5
**10-14**	0.79 (0.77, 0.81)	18.3	0.58	80.0	77.9	16.3	98.6	77.9
**15-19**	0.84 (0.80, 0.88)	20.2	0.68	78.4	89.8	12.4	99.6	89.6
